# Identifying the clinical signature of anti-centromere antibody-positive Sjögren’s syndrome: a machine learning-based analysis of a multicenter cohort

**DOI:** 10.3389/fimmu.2026.1803065

**Published:** 2026-04-23

**Authors:** Wenlong Zhu, Ting Fang, Xinchao Zhu, Zhe Yang, Jiayao Wang, Xinchang Wang

**Affiliations:** 1 The Second Clinical Medical College, Zhejiang Chinese Medical University, Hangzhou, China; 2 Department of Rheumatology, The Second Affiliated Hospital of Zhejiang Chinese Medical University, Hangzhou, China

**Keywords:** anti-centromere antibody, autoantibodies, machine learning, SHAP algorithm, Sjögren’s syndrome

## Abstract

**Objective:**

We sought to leverage machine learning algorithms to identify the complex clinical and serological signature of anti-centromere antibody (ACA) positivity in Sjögren’s syndrome (SS) patients.

**Methods:**

This multicenter study analyzed clinical data from a cohort of 616 patients diagnosed SS, comprising 81 ACA-positive and 535 ACA-negative cases. To ensure robust model development, we randomly partitioned the dataset into training and validation subsets in a 7:3 ratio. We implemented and compared six machine learning models after identifying optimal predictors using the LASSO regression. We mainly evaluate the performance of the model through the AUC and a series of comprehensive indicators. To ensure clinical interpretability, we also employed the SHAP analysis method to quantify the influence of each feature on the model’s outcome.

**Results:**

Among the evaluated models, GBDT exhibited superior predictive efficacy. The model achieved an AUC value of 0.812 in the training set and maintained a robust AUC of 0.811 (95% CI: 0.699–0.906) in the validation cohort. At the same time, the model has the highest sensitivity (0.750 in the validation test). The SHAP analysis revealed that the top predictors influencing the ACA-positive profile included a series of serological markers (anti-SSA/Ro52, anti-SSA/Ro60, anti-AMA-M2, anti-SSB, and IgM), demographic factors (age), and Raynaud’s phenomenon (RP). Furthermore, SHAP interactions captured non-linear synergies, such as the predictive contribution of RP is significantly potentiated by advancing age, and the amplified predictive value of anti-AMA-M2 under lower IgM levels.

**Conclusion:**

Our machine learning approach effectively structures and quantifies clinical and serological associations, capturing a complex predictive profile for the SS-ACA^+^ subgroup. These findings highlight the value of ML in identifying non-linear patterns within clinical variables, providing a robust quantitative framework for future prospective evaluations.

## Introduction

Sjögren’s syndrome (SS) is a chronic autoimmune disorder primarily defined by immune-mediated dysfunction of the exocrine glands. The pathognomonic features remain xerostomia and xerophthalmia, but the clinical spectrum of SS is notably heterogeneous. Beyond sicca symptoms, patients also present with a broad array of extraglandular manifestations, ranging from Raynaud’s phenomenon (RP) and neurological disorders (1, 2). Notably, SS can also affect multiple organs, such as the lungs, skin and liver (3, 4).According to the current ACR/EULAR classification criteria for SS, the diagnosis of SS hinges on the presence of objective xerostomia and/or xerophthalmia, accompanied by evidence of either histologically proven lymphocytic foci in salivary gland biopsies or the presence of serum anti-SSA/Ro autoantibodies (5).Although anti-centromere antibodies (ACAs) are not currently included in the diagnostic or classification criteria for SS, their potential association with sicca symptoms warrants clinical awareness.

ACA is a class of autoantibodies targeting centromere-associated proteins during cell division, and belong to a subtype of antinuclear antibodies (ANA) (6, 7).ACA is linked to several autoimmune disorders and are commonly identified in patients with systemic sclerosis (SSc) and primary biliary cholangitis (PBC) in clinical settings (8). Studies have reported a prevalence of ACA ranging from 3% to 15% among patients with SS. ACA-positive patients confer an elevated risk of subsequent development of PBC or SSc, and are also characterized by a higher frequency of RP in clinical manifestations (9, 10).

In recent years, machine learning (ML) algorithms have exhibited remarkable performance improvements in the medical field, demonstrating distinct advantages in a wide range of clinical and research applications (11).More specifically, ML has shown tremendous potential in the prediction and diagnosis of diseases within the field of rheumatology and immunology (12, 13). ML algorithms represent a transformative approach to medical informatics, capable of orchestrating extensive multi-faceted data into actionable predictive outputs. This methodology not only enhances the accuracy of diagnosis and effectively reduces misclassification, but also facilitates the formulation of individualized medical plans. Compared with traditional linear statistical methods, ML can identify potential early risks and provide solid data support for personalized intervention measures (14).

This study developed multiple ML models integrating demographic characteristics, clinical symptoms, and laboratory indicators to identify the clinical profile of the ACA-positive subgroup in SS patients and to evaluate the optimal model.

## Materials and methods

This study was approved by the Institutional Ethics Committee of The Second Affiliated Hospital, Zhejiang Chinese Medical University (No. 2023-KL-003-A01). The studies were conducted in accordance with the local legislation and institutional requirements. All participants provided written informed consent for future research use of their data.

### Study population

This study included data from a prospective cohort comprising 931 patients diagnosed with SS who presented between June 2023 and October 2025. These clinical data were obtained from the Second Affiliated Hospital of Zhejiang Chinese Medical University (n=236), the First Affiliated Hospital of Zhejiang Chinese Medical University (n=84), the First Affiliated Hospital of Zhejiang University School of Medicine (n=45), the Second Affiliated Hospital of Zhejiang University School of Medicine (n=242), Sir Run Shaw Hospital Affiliated with Zhejiang University School of Medicine (n=176), Huzhou Third People’s Hospital (n=21), Jiaxing Second Hospital (n=59), and Shaoxing Hospital of Traditional Chinese Medicine (n=68). All participants conformed to the 2016 ACR/EULAR classification criteria for SS (5). Patients who met the 2013 ACR/EULAR classification criteria for systemic sclerosis (15), or fulfilled the 2018 American Association for the Study of Liver Diseases (AASLD) or the European Association for the Study of the Liver (EASL) diagnostic criteria for active PBC were rigorously excluded from the study (16).

After a screening process, 616 patients met the inclusion criteria and were retained for analysis. The analytical dataset consisted of 81 individuals with SS-ACA^+^ and 535 with SS-ACA^-^.

### Data collection

Clinical data collection is mainly divided into three main areas: basic demographic information, major clinical manifestations, and involvement of systemic organs. Demographic data captured age, sex, height, weight, disease duration (from initial symptom onset to definitive diagnosis), and age at onset. The spectrum of clinical manifestations encompassed sicca symptoms (xerostomia and xerophthalmia), arthralgia, RP, rampant caries, and parotid gland enlargement. To assess whether each organ was affected, we systematically evaluated the involvement of the hematological system, the lungs, the skin, the liver and the kidneys. In addition, the degree of disease activity was quantitatively evaluated using the EULAR Sjögren’s Syndrome Disease Activity Index (ESSDAI) and EULAR Sjögren’s Syndrome Patient Reported Index (ESSPRI).

Laboratory assessments mainly covered the following parameters: white blood cell count (WBC), neutrophil count (NE), lymphocyte count (LY), red blood cell count (RBC), hemoglobin level (HGB), platelet count (PLT), erythrocyte sedimentation rate (ESR), alanine aminotransferase (ALT), aspartate aminotransferase (AST), total protein (TP), globulin (GLO), albumin (ALB), total bilirubin (TBIL), indirect bilirubin (IBIL), creatinine (CREA), gamma-glutamyl transferase (GGT), alkaline phosphatase (ALP), C-reactive protein (CRP), anti-SSA/Ro60 antibody, anti-SSA/Ro-52 antibody, anti-SSB/La antibody, anti-Jo-1 antibody, anti-U1RNP antibody, anti-Scl-70 antibody, ACA, anti-ds-DNA antibody, anti-AMA-M2 antibody, IgA, IgG, IgM, C3, and C4.

### Statistical analysis

Computational modeling and data integration were performed using Python (v3.8) and R (v4.3.3). The machine learning framework was implemented primarily within the scikit-learn ecosystem. Baseline characteristics were summarized and compared across cohorts using the ‘CBCgrps’ package in R. Categorical variables were evaluated through the χ (2) test and presented in the form of frequencies (%). Continuous data with non-normal distribution were analyzed using the Mann-Whitney U test and reported in the form of the median and its interquartile range (IQR). Variables with a missing data rate exceeding 30% were excluded from the analysis. For the remaining variables, we implemented Multiple Imputation by Chained Equations (MICE) to mitigate potential selection bias and preserve the statistical power of our multicenter clinical dataset. Statistical significance was defined by a two-tailed P-value < 0.05. To determine the most concise set of predictive factors, we employed the LassoCV method and combined it with 10-fold cross-validation to determine the optimal regularization parameter (λ), retaining the features with non-zero coefficients. The dataset was randomly partitioned into training (70%) and validation (30%) subsets. To systematically benchmark predictive performance, these algorithms were purposefully selected to represent conventional linear modeling (LR), bagging ensemble techniques (RF), and advanced gradient boosting architectures (GBDT, AdaBoost, XGBoost, and LightGBM). Through random search cross validation and 5 fold cross-validation of systemic parameter adjustment process, the model structure is optimized. We evaluated the performance of the model by conducting a rigorous assessment of the AUC, sensitivity, specificity, accuracy, F1 value, Brier scores and 95%CI. Detailed configurations and the optimization process for the model’s hyperparameters are provided in the **Supplementary Materials**.

### SHAP algorithm

To enhance the interpretability of the predictive model, we integrated the SHAP analysis to decode the underlying decision-making logic. We used the shap Python library (version 0.40.0) to calculate the Shapley values, in order to precisely quantify the marginal contribution of each diagnostic feature to the specific risk assessment. This method can identify the most influential clinical predictors. Then construct a multi-dimensional SHAP visualization graph to clearly demonstrate the degree and direction of the correlation between features and results, thereby presenting the model’s outcomes in an intuitive manner.

## Results

### Baseline characteristics

We identified 616 patients with SS, of whom 81 (13.2%) in the ACA^+^ group and 535 (86.8%) in the ACA^-^ group. The overall median age was 56 years, and the ACA^+^ group was significantly older (P < 0.001). Serological analysis revealed higher rates of anti−AMA−M2 antibodies in the ACA^+^ group (all P < 0.001), whereas anti−SSA/Ro60, anti−SSA/Ro52, and anti−SSB positivity was lower (P < 0.001). Furthermore, the ACA^+^ group exhibited significantly reduced levels of globulin and IgG (both P < 0.01). Clinically, RP was more frequent in the ACA^+^ group (P < 0.001), while no significant differences were observed in the prevalence of dry mouth, dry eye, or interstitial lung disease (all P > 0.05). Additionally, concomitant primary biliary cholangitis was more common in the ACA^+^ group (P = 0.005) (**Table 1**).

**Table 1:** Comparison of various parameters between SS patients.

Variables	Total (n = 616)	ACA-negativity (n = 535)	ACA-positivity (n = 81)	p	statistic
anti-SSA/Ro60, n (%)				< 0.001	37.484
negativity	102 (17)	69 (13)	33 (41)		
positivity	514 (83)	466 (87)	48 (59)		
anti-SSA/Ro52, n (%)				< 0.001	30.699
negativity	127 (21)	91 (17)	36 (44)		
positivity	489 (79)	444 (83)	45 (56)		
anti-SSB, n (%)				< 0.001	12.361
negativity	357 (58)	295 (55)	62 (77)		
positivity	259 (42)	240 (45)	19 (23)		
anti-jo1, n (%)				0.345	Fisher
negativity	613 (100)	533 (100)	80 (99)		
positivity	3 (0)	2 (0)	1 (1)		
anti-U1RNP, n (%)				1	Fisher
negativity	582 (94)	505 (94)	77 (95)		
positivity	34 (6)	30 (6)	4 (5)		
anti-Scl70, n (%)				1	Fisher
negativity	611 (99)	530 (99)	81 (100)		
positivity	5 (1)	5 (1)	0 (0)		
anti-ds-dna, n (%)				0.408	Fisher
negativity	587 (95)	508 (95)	79 (98)		
positivity	29 (5)	27 (5)	2 (2)		
anti-AMA-M2, n (%)				< 0.001	16.814
negativity	554 (90)	492 (92)	62 (77)		
positivity	62 (10)	43 (8)	19 (23)		
sex, n (%)				0.408	Fisher
female	587 (95)	508 (95)	79 (98)		
male	29 (5)	27 (5)	2 (2)		
dry-mouth, n (%)				0.058	3.586
negativity	129 (21)	119 (22)	10 (12)		
positivity	487 (79)	416 (78)	71 (88)		
dry-eye, n (%)				0.351	0.868
negativity	191 (31)	170 (32)	21 (26)		
positivity	425 (69)	365 (68)	60 (74)		
ILD, n (%)				0.468	0.526
negativity	571 (93)	498 (93)	73 (90)		
positivity	45 (7)	37 (7)	8 (10)		
Raynaud, n (%)				< 0.001	81.391
negativity	562 (91)	510 (95)	52 (64)		
positivity	54 (9)	25 (5)	29 (36)		
wbc-low, n (%)				0.331	0.946
negativity	513 (83)	442 (83)	71 (88)		
positivity	103 (17)	93 (17)	10 (12)		
plt-low, n (%)				0.678	0.173
negativity	567 (92)	491 (92)	76 (94)		
positivity	49 (8)	44 (8)	5 (6)		
PBC, n (%)				0.005	Fisher
negativity	602 (98)	527 (99)	75 (93)		
positivity	14 (2)	8 (1)	6 (7)		
age, years (Q1,Q3)	56 (46, 62)	55 (44, 62)	61 (55, 67)	< 0.001	14872.5
weight, kg (Q1,Q3)	55 (50, 61.12)	55 (50, 62)	56 (50, 60.5)	0.998	21672.5
height, cm (Q1,Q3)	160 (156, 163)	160 (156, 163)	159 (156, 162)	0.24	23414
ESSPRI, scores (Q1,Q3)	3.67 (2.33, 4.75)	3.67 (2.33, 4.67)	4 (2.67, 5.33)	0.08	19059
WBC, 10^9/L (Q1,Q3)	4.7 (3.8, 6)	4.61 (3.8, 5.99)	4.9 (4, 6.1)	0.223	19848
NE, 10^9/L (Q1,Q3)	2.66 (2, 3.72)	2.61 (2, 3.7)	2.8 (2.2, 3.8)	0.204	19769.5
LY, 10^9/L (Q1,Q3)	1.48 (1.11, 1.9)	1.5 (1.12, 1.9)	1.4 (1.1, 1.73)	0.497	22680.5
RBC, 10^12/L (Q1,Q3)	4.2 (3.91, 4.45)	4.21 (3.91, 4.48)	4.13 (3.89, 4.35)	0.257	23359.5
HGB, g/L (Q1,Q3)	126 (118, 133)	126 (117.7, 134)	124 (118, 131)	0.481	22718.5
PLT, 10^9/L (Q1,Q3)	192 (157, 232.25)	193 (160, 233.5)	174 (149, 218)	0.122	23973.5
ESR, mm/h (Q1,Q3)	19 (9, 35)	19 (9, 34.5)	15 (8, 35)	0.158	23773
ALT, IU/L (Q1,Q3)	18 (13, 25.7)	18 (13, 25)	18 (13, 26)	0.966	21604
AST, IU/L (Q1,Q3)	22.15 (19, 29)	22 (18.6, 29)	25 (20, 30)	0.276	20043
TP, g/L (Q1,Q3)	75.8 (71.26, 79.93)	76 (71.65, 80.1)	73.7 (67.7, 78)	0.002	26297
GLO, g/L (Q1,Q3)	33.5 (29.73, 37.2)	34 (30, 37.35)	30.5 (27.9, 35.1)	0.001	26500.5
ALB, g/L (Q1,Q3)	42 (39.4, 44.1)	42.1 (39.4, 44.1)	41.9 (39.5, 43.5)	0.486	22707
TBIL, µmol/L (Q1,Q3)	11.3 (9, 14.4)	11.1 (8.9, 14.2)	12.1 (9.6, 16.2)	0.036	18531.5
IBIL, µmol/L (Q1,Q3)	8.3 (6.2, 11.1)	8 (6, 10.95)	9.5 (7.2, 12.3)	0.013	17952.5
CREA, µmol/L (Q1,Q3)	59.45 (52.98, 66.3)	59.2 (52.6, 66)	61 (53.9, 67)	0.504	20670
GGT, IU/L (Q1,Q3)	18 (14, 30)	17 (13.6, 28)	21 (14, 37)	0.034	18502
ALP, IU/L (Q1,Q3)	69 (57, 87)	69 (56, 86)	72 (62, 90)	0.045	18681.5
CRP, mg/L (Q1,Q3)	1.7 (0.8, 3.6)	1.6 (0.8, 3.66)	1.8 (0.9, 3.2)	0.694	21080.5
IgA, g/L (Q1,Q3)	2.96 (2.26, 4.01)	2.96 (2.29, 4.01)	3.04 (1.83, 3.87)	0.278	23286
IgM, g/L (Q1,Q3)	1.21 (0.89, 1.66)	1.21 (0.9, 1.63)	1.39 (0.84, 2.05)	0.181	19672.5
IgG, g/L (Q1,Q3)	16.26 (13.5, 19.92)	16.73 (13.7, 20.18)	14.14 (11.8, 16.55)	< 0.001	28268
C3, g/L (Q1,Q3)	0.94 (0.78, 1.11)	0.93 (0.77, 1.11)	0.96 (0.81, 1.16)	0.278	20047.5
C4, g/L (Q1,Q3)	0.24 (0.17, 1.66)	0.23 (0.17, 1.64)	0.27 (0.19, 1.77)	0.202	19764
ESSDAI, scores (Q1,Q3)	2 (1, 5)	2 (1, 5)	2 (0, 5)	0.149	23801
group, n (%)				1	0
test	184 (30)	160 (30)	24 (30)		
train	432 (70)	375 (70)	57 (70)		

WBC, white blood cell count; NE, neutrophil count; LY, lymphocyte count; RBC, red blood cell count; HGB, hemoglobin level; PLT, platelet count; ESR, erythrocyte sedimentation rate; ALT, alanine aminotransferase; AST, aspartate aminotransferase; TP, total protein; GLO, globulin; ALB, albumin; TBIL, total bilirubin; IBIL, indirect bilirubin; CREA, creatinine; GGT, gamma-glutamyl transferase; ALP, alkaline phosphatase; CRP, C-reactive protein; PBC, primary biliary cholangitis; ILD, Interstitial Lung Disease; ESSDAI, EULAR Sjögren’s Syndrome Disease Activity Index; ESSPRI, EULAR Sjögren’s Syndrome Patient Reported Index.

### Association between clinical, serological, and laboratory features in patients with SS

**Figure 1:**
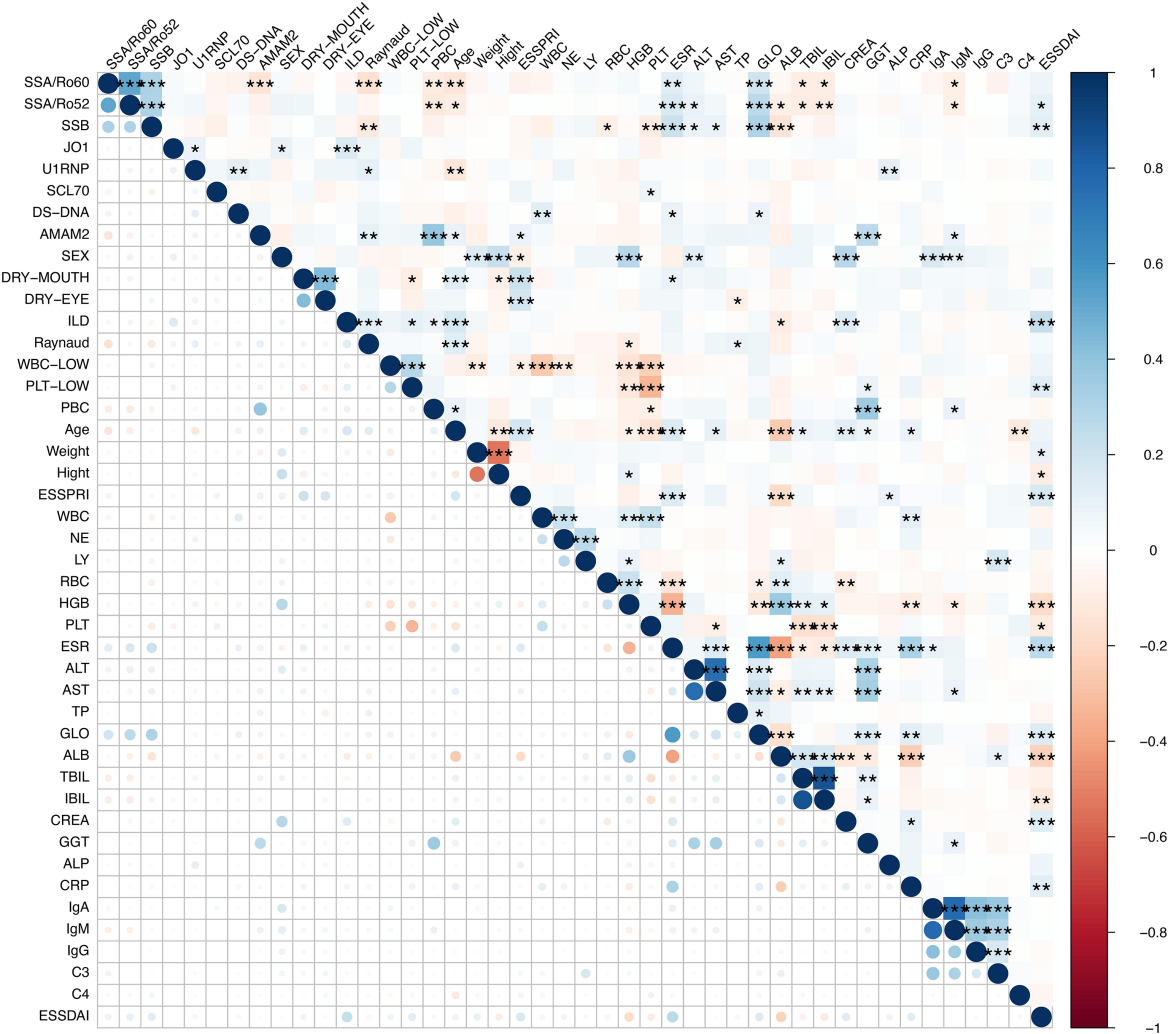
Correlation analysis between Clinical, Serological, and Laboratory Features in Patients with SS. Asterisks denote the level of statistical significance of the correlation coefficients ( *P < 0.05, ** P < 0.01, *** P < 0.001).

We further investigated the associations among clinical, serological, and laboratory features in patients with SS. The results indicated that serum-specific autoantibodies (SSA/Ro, SSB) were correlated with both typical exocrine gland manifestations (dry mouth, dry eye) and systemic involvement (interstitial lung disease, RP). Moreover, inflammatory markers (ESR, CRP) and immunoglobulin levels (IgG, IgA, IgM) showed significant correlations with disease activity (ESSDAI), while C3 and C4 were associated with certain activity indicators. Hematological abnormalities, including leukopenia and thrombocytopenia, also exhibited correlations with immune activation parameters. These findings collectively delineate the interconnected landscape of immune dysregulation and clinical manifestations in SS (**Figure 1**).

### Feature selection

**Figure 2:**
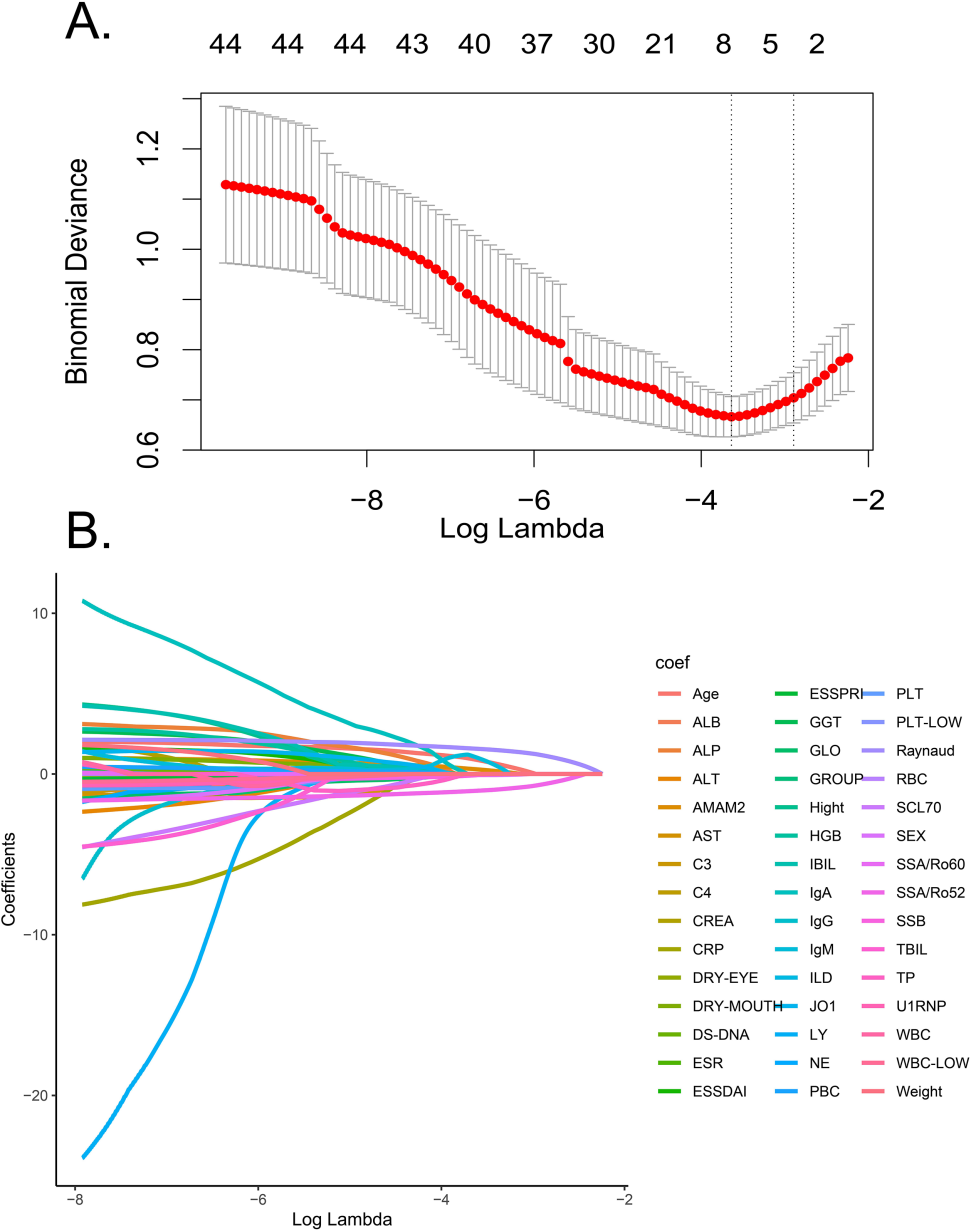
LASSO regression analysis for feature selection. **(A)** Cross-validated deviance curve against log (λ), where λ is the regularization parameter; vertical dotted lines indicate optimal λ values, identifying 7 main features for the model; **(B)** shows the LASSO coefficient paths for 45 features. LASSO, Least Absolute Shrinkage and Selection Operator.

We employed the LASSO regression method to optimize the variable set and reduce the potential overfitting phenomenon. This algorithm effectively compresses the coefficients with less information to absolute zero values by incorporating the L1 penalty term into the objective function, thereby simplifying the prediction architecture. The LASSO regression analysis included 45 variables from the training set, and the model selection was accomplished using the 10-fold cross-validation method (**Figure 2A**). The regularization dynamics of the 45 candidate predictors were visualized through LASSO coefficient profiles (**Figure 2B**). The λ was optimized through cross-validation, and ultimately seven features with significant influence were screened out from the original dataset. Specifically, age, the positive rate of autoantibodies related to SS (anti SSA/Ro60, anti SSA/Ro52, anti SSB), the positive rate of anti AMA-M2, RP, and IgM levels showed the strongest correlations.

### Model evaluation

**Figure 3:**
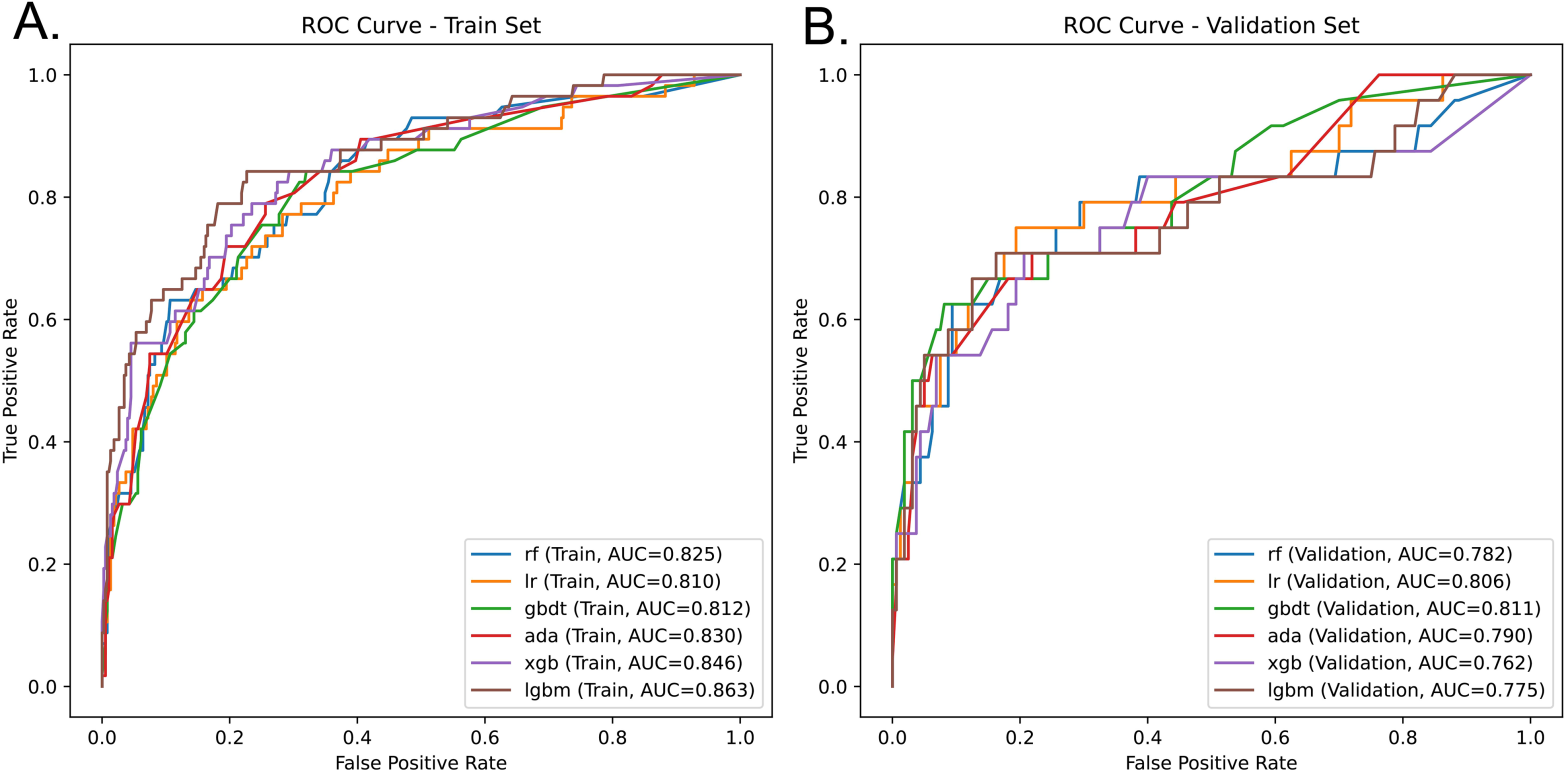
Performance evaluation of six machine learning classification algorithms. **(A)** ROC curves of algorithms in the train set; **(B)** ROC curves of algorithms in the validation set. ROC, receiver operating characteristic; AUC, Area Under the Curve; RF, Random Forest; LR, Logistic Regression; GBDT, Gradient Boosting Decision Tree; ADA, Adaptive Boosting; XGB, Extreme Gradient Boosting; LGBM, Light Gradient Boosting Machine.

We trained six models (RF, LR, GBDT, ADA, XGBoost, and LGBM) using the 10-fold cross-validation method and conducted evaluations (**Supplementary Table 1**). The comparative analysis of the ROC curves of the training and validation set indicates that GBDT is determined to be the optimal model. In the training set, the AUC of the LGBM algorithm reached 0.863 (95% CI: 0.802 - 0.914). GBDT also performed well with an AUC value of 0.812 (95% CI: 0.745-0.870) (**Figure 3A**). Notably, both models yielded a peak sensitivity of 0.842, which highlights their strong discriminatory ability. Among all the models, the AUC value of the validation set generally showed a certain degree of decline. We also conducted the DeLong test, and the results showed that there was no significant statistical difference (**Supplementary Table 2**). However, the GBDT demonstrated remarkable stability, with its AUC reaching the highest value of 0.811 (95% CI: 0.699 - 0.906). Meanwhile, GBDT achieved the optimal sensitivity of 0.750. Although the LGBM showed excellence during training set, it suffered a significant performance decay on the validation set (AUC = 0.775) (**Figure 3B**). Threshold optimization was conducted based on the Youden index to equilibrate the model’s predictive metrics (**Supplementary Table 3**). The predictive integrity and clinical utility of the GBDT model were further appraised through calibration curves (**Supplementary Figure 1**) and decision curve analysis (DCA) (**Supplementary Figure 2**). Superior predictive accuracy and consistent performance stability validated GBDT as a reliable tool with enhanced discriminative capacity compared to other models.

### Feature importance based on the SHAP analysis

**Figure 4:**
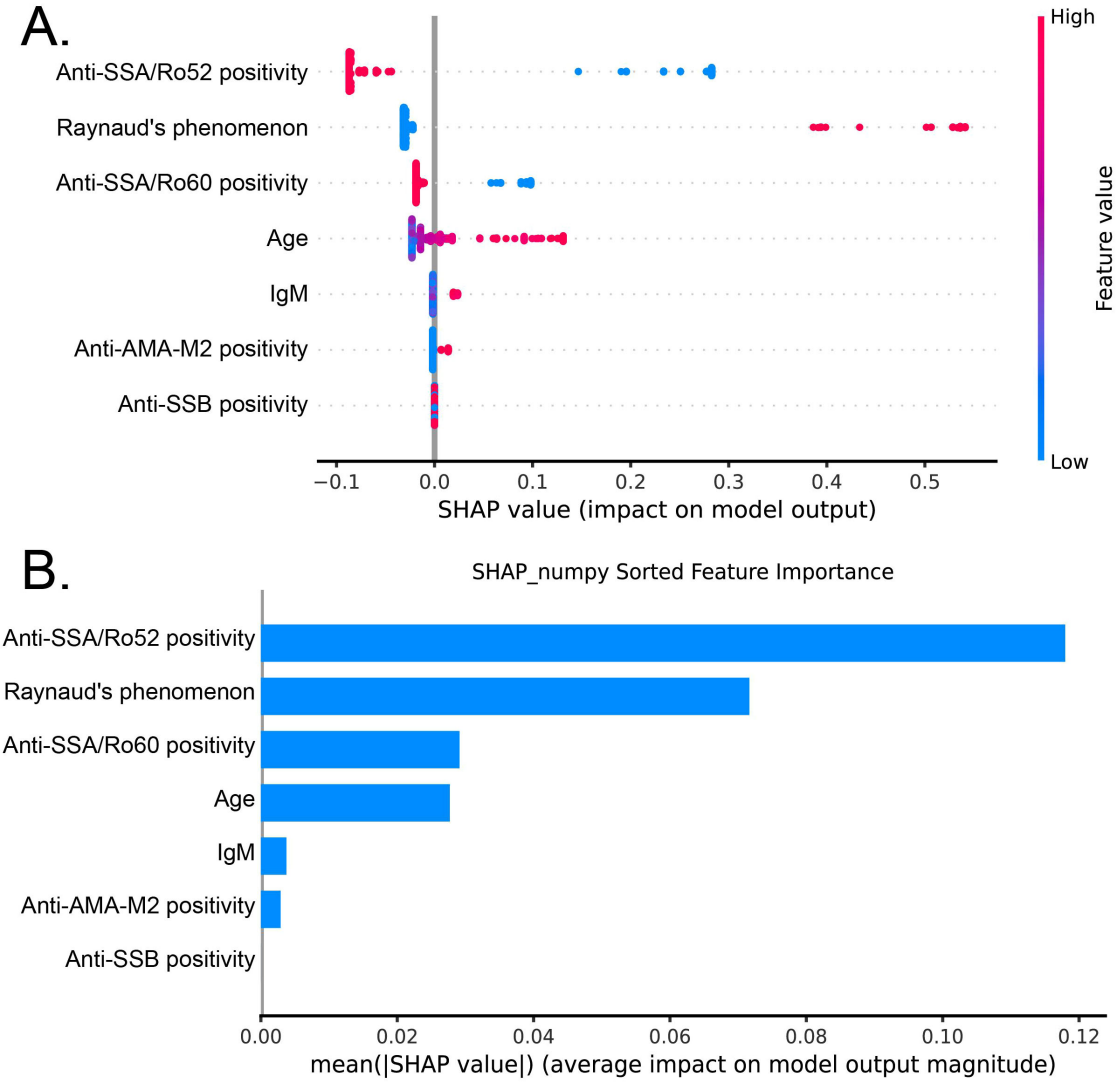
SHAP analysis of feature importance in the GBDT algorithm. **(A)** Distribution of SHAP values for each feature, demonstrating the impact of individual predictors on model output. Each point represents a single observation, with color indicating feature value; **(B)** Mean absolute SHAP values quantifying the average magnitude of feature contributions to model outputs, arranged in descending order of importance. SHAP, SHapley Additive exPlanations.

We employed the SHAP value analysis method to quantify the contribution and directional impact of specific clinical and immunological characteristics. Our findings identified the positivity of anti-SSA/Ro52 antibodies as the most significant indicator. The positive rates of anti-SSA/Ro52 and anti-SSA/Ro60 were both negatively correlated with the target outcome, underscoring the prevalence of these antibodies is usually lower in SS-ACA^+^ patients. Conversely, the presence of RP is positively correlated with the prediction results and becomes a powerful indicator influencing the outcome. While features such as age, IgM levels, anti-AMA-M2 and anti-SSB status also contributed to the model’s output, their relative influence was comparatively modest. (**Figure 4A**).

We calculated the average absolute SHAP value to quantify the relative importance of each predictor. The positive rate of anti-SSA/Ro52 antibodies became the main determining factor. Its average SHAP value was significantly higher than that of other variables, further confirming its status as the most crucial feature in the model. Meanwhile, the presence of RP and the positivity of anti-SSA/Ro60 antibodies also have significant value. In comparison, variables such as age, serum IgM levels, positivity for anti-AMA-M2 and anti-SSB antibodies exerted relatively minor effects on the classification of the SS-ACA^+^ patients (**Figure 4B**).

We further evaluated feature interactions using SHAP dependence plots to decode the complex relationships within the model. The results unveiled a significant synergistic interaction between RP and age (**Supplementary Image 1A, B**). Specifically, the contribution of RP was modulated by age, with its effect being markedly potentiated in elderly patients. Furthermore, we identified a non-linear synergistic interaction between anti-AMA-M2 positivity and serum IgM levels (**Supplementary Image 1C, D**). Notably, the association of anti-AMA-M2 with the target subset was significantly amplified at lower IgM thresholds.

## Discussion

SS is a systemic autoimmune epithelitis primarily defined by exocrine insufficiency and a hallmark serological manifestations. Anti-SSA/Ro antibodies and anti-SSB antibodies are crucial biomarkers within the existing SS diagnostic framework. The former are typically classified as Ro52 and Ro60 protein types in clinical laboratory assays. However, the clinical and serological features of SS go far beyond these typical indicators. Although ACA has not been included in the official classification criteria, it defines a unique clinical subtype. Studies suggested that ACA positivity occurs in 3% to 15% of the SS population, representing a unique endotype that warrants deeper phenotypic characterization (17–19). Of clinical significance, ACA positivity is firmly associated with an increased risk of concomitant autoimmune pathologies, especially the convergence of SS with SSc and PBC. This association also extends to the vascular domain, where RP is a typical symptom manifestation (20, 21). SS-ACA^+^ patients frequently present with associated clinical features. Prioritizing the early detection of this serological subgroup may assist in clinical monitoring.

ACA is a subtype of ANA and represents a significant component of the ANA profile. ACA primarily targets centromere proteins (CENPs), including CENP-A, CENP-B, and CENP-C, and may occasionally react with heterochromatin protein 1α (HP1α). It is widely recognized as a serological marker for the diagnosis of SSc (22, 23). SSc is a complex multisystemic autoimmune disorder characterized by involvement of the skin and internal organs. Studies have shown that the development of pulmonary fibrosis is frequently observed and is strongly associated with an unfavorable prognosis in SSc cohorts (24, 25). A review study indicated that up to 80% of SSc patients present with orofacial manifestations such as xerostomia and xerophthalmia, which are hypothesized to be associated with salivary gland fibrosis. Moreover, ACA-positive SSc patients are more likely to exhibit a higher propensity to develop sicca symptoms (26). As the detection rate of ACA in the serum of patients with autoimmune rheumatic diseases other than SSc has increased, seroimmunological investigations have elucidated discrete variations in CENP-recognition patterns between SS and SSc cohorts (27). This suggests a speculative hypothesis that the immunopathological driving factors for the two diseases may originate from different mechanisms. These results serve as a reminder for doctors to remain vigilant about the possibility of overlapping symptoms in the group of SS-ACA^+^ patients.

Patients in the SS-ACA^+^ subgroup frequently present with a broad spectrum of extraglandular manifestations, with a notably higher association with hepatic system involvement. Indeed, the reported 20%-30% prevalence of concurrent SS among PBC cohorts underscores the high degree of immunological overlap between these two conditions (28). Our analysis identified anti-AMA-M2 seropositivity and elevated serum IgM levels as robust predictive features associated with the ACA-positive subgroup in patients with SS. Anti-mitochondrial antibodies (AMAs), particularly the M2 subtype, are the specific serological markers of PBC. The diagnostic specificity and positive rate of this antibody in this population exceed 90% (29, 30). As a cornerstone of the PBC diagnostic framework, AMA exhibits unparalleled specificity and is characteristically coupled with elevated IgM levels (31). To date, establishing validated surrogate biomarkers for AMA-negative PBC continues to be an active area of clinical inquiry (32). Notably, ACA is detectable in both primary SS and PBC, suggesting their potential role as a serological marker of an overlap syndrome, such as concurrent SS and PBC (20, 33). Emerging literatures have found that the positive characteristics of ACA are associated with a tendency towards fibrotic transformation in SS patients. The presence of ACA appears to function as a deleterious prognosticator in PBC population, potentially heralding a more aggressive disease course (4, 34).

Compared with the ACA-negative patient group, SS-ACA^+^ patients exhibit a unique combination of clinical features. Beyond the characteristic sicca symptoms, this subset is also associated with an older age of onset, RP and the gastrointestinal tract (35–37). These findings are consistent with our results that RP and older age are prominent clinical manifestations for SS-ACA^+^. RP serves as a critical clinical sentinel for systemic vasculopathy, predisposing patients with autoimmune rheumatic diseases to cardiovascular disorders (38, 39). Studies showed that the SS-ACA^+^ patients are more prone to left ventricular diastolic dysfunction, a condition that may insidiously progress to overt heart failure and emerges as a primary determinant of mortality in this cohort (40). These observations remind clinicians to remain alert to RP when evaluating patients with SS, particularly within the ACA-positive subgroup.

ML algorithms are increasingly being integrated into rheumatology to refine diagnostic accuracy and prognostic stratification (41, 42). In the current study, we leveraged ML frameworks to delineate the specific clinical signature of ACA-positive SS patients. Among the six evaluated machine learning algorithms, the GBDT model consistently outperformed other candidates. Its AUC values in the training and validation set were 0.812 (95% CI: 0.745 - 0.870) and 0.811 (95% CI: 0.699 - 0.906), respectively. The consistent peak sensitivity (0.842 and 0.750) further confirm their clinical discriminative ability. To mitigate multicollinearity and confounding, we employed LASSO regression for feature selection. Next came the attribution analysis based on SHAP, which identifies a hierarchical seven-factor model. Within this framework, the positive rate of anti-SSA/Ro52, RP, and anti-SSA/Ro60 status were identified as the top predictive features contributing to the model output.

From a clinical immunology standpoint, anti-SSA/Ro and anti-SSB antibodies constitute the serological cornerstone of SS. Anti-SSA/Ro antibodies are usually divided into two subtypes: anti-SSA/Ro52 and anti-SSA/Ro60. The prevalence of these markers, especially anti-SSA/Ro, in the SS-ACA^+^ patients was significantly lower than that in the ACA-negative subgroup (9, 36). This trend is in complete agreement with the hierarchical feature importance that we identified in the SHAP-based analysis. A study conducted in South Korea involving 318 SS patients highlighted similar phenotypic differences (21). It was found that ACA-positivity was intrinsically linked to a higher incidence of RP and hepatic dysfunction, yet inversely associated with the classic anti-SSA/Ro profile. These concordant results validate our predictive factors and emphasize the necessity of broad-spectrum autoantibody screening for precise patient stratification.

Intriguingly, our SHAP interaction analysis unveiled a significant non-linear synergy between RP and age, characterized by a markedly elevated contribution of RP in elderly cohorts. This interaction suggests that as disease duration extends with age, vascular involvement may transition from a transient clinical manifestation into a stable phenotypic signature. Consequently, RP emerges as a highly specific clinical anchor for the SS-ACA^+^ subgroup in older patients. This synergistic logic effectively reduces non-specific RP background noise prevalent in younger populations and underscores the necessity for clinicians to maintain high vigilance for ACA positivity and underlying systemic involvement in elderly patients presenting with new-onset SS and RP. Furthermore, we identified a nuanced non-linear interaction between anti-AMA-M2 positivity and serum IgM levels. The contribution of anti-AMA-M2 was markedly potentiated in the context of lower IgM levels. This interaction suggests that anti-AMA-M2 may act as a critical diagnostic anchor for the SS-ACA^+^ subgroup, particularly when generalized humoral immune activation is less prominent. For these atypical serological cases, clinicians should maintain high vigilance for underlying visceral involvement, even in the absence of overt systemic inflammatory markers.

While conventional statistical algorithms have historically provided a foundation for clinical analysis, they often lack the granularity required to navigate the multidimensionality and non-linear intricacies inherent in complex autoimmune datasets. Specifically, traditional linear models struggle to capture conditional dependencies. Our machine learning approach provides critical incremental value by decoding non-linear dynamics. Coupled with SHAP interaction analysis, GBDT model revealed that the predictive importance of features is not static. For instance, the importance of RP is synergistically amplified by age, and anti-AMA-M2 positivity exhibits a profound contribution precisely when systemic IgM levels are relatively low. This method boasts excellent computational efficiency and is capable of simulating high-order interactions. Leveraging a comprehensive multicenter database covering various parameters, this study systematically benchmarked six ML candidates. The GBDT emerged as the most resilient model. As an ensemble learning paradigm, GBDT excels in non-linear optimization and generalizability. Its inherent interpretability provides a unique diagnostic perspective for identifying the complex clinical features of SS-ACA^+^ patients.

Despite its clinical insights, this study possesses inherent constraints that merit acknowledgment. Given the inherent rarity of the clinical subset under study, we pooled multicenter data from Zhejiang Province to maximize statistical power and ensure that the machine learning models could capture a sufficiently diverse range of features. We acknowledge that the lack of an independent external validation cohort from a different geographic region is a limitation. However, our current findings provide a statistically sound and clinically relevant foundation for future prospective, multi-regional validation studies. In order to reinforce the reliability of ML results, it will be necessary to conduct verification in larger-scale and more diverse data cohorts in the future. Our analysis is mainly based on clinical manifestations and serological parameters, offering a focused but singular observational lens. SHAP values quantify the mathematical contribution of specific features to the model’s predictions, and they do not establish biological causality or mechanistic pathways. The non-linear interactions identified in our study should be interpreted strictly as algorithmic predictive synergies rather than direct pathophysiological links, warranting further basic science research. Integrating multi-omics data for analysis in the future is a reasonable next step to reveal the complex mechanism pathways underlying this specific subset. Finally, while our methodology aligned with the 2016 ACR/EULAR criteria. It is important to note that future updates to these classification criteria could introduce label shifts within patient cohorts. Such evolving diagnostic frameworks would necessitate the recalibration of our ML model to ensure its continued predictive validity in real-world clinical settings.

## Conclusion

To conclude, our findings demonstrate that the GBDT model provides a robust internal framework for identifying the clinical associations of ACA-positive SS. Our ML results identified serum positivity rate of anti-SSA/Ro52 and anti-SSA/Ro60, RP, age at onset, IgM levels, anti-AMA-M2 and anti-SSB reactivity as the key predictive features of this subset. SHAP interaction analysis also captured complex non-linear synergies within the model, notably between RP and age, as well as between anti-AMA-M2 positivity and serum IgM levels.

## Data Availability

The datasets presented in this study can be found in online repositories. The names of the repository/repositories and accession number(s) can be found below: GitHub:https://github.com/2085559633/SS-ACA-ML.git.

## Supplementary material

Supplementary Figure 1 Comparative calibration curves of the six evaluated machine learning algorithms in the validation cohort.

Supplementary Figure 2 Decision curve analysis (DCA) evaluating the clinical utility of the six machine learning models in the validation cohort.

Supplementary Figure 3 SHAP dependence and interaction plots for key features. **(A–B)** SHAP interaction between RP and age. **(C–D)** SHAP interaction between anti-AMA-M2 positivity and serum IgM level.
