# Supplementary figures and images for: Identifying the clinical signature of anti-centromere antibody-positive Sjögren’s syndrome: a machine learning-based analysis of a multicenter cohort
Source: Front Immunol. 2026 Apr 23;17:1803065. doi: 10.3389/fimmu.2026.1803065 (PMC13149254; doi:10.3389/fimmu.2026.1803065)

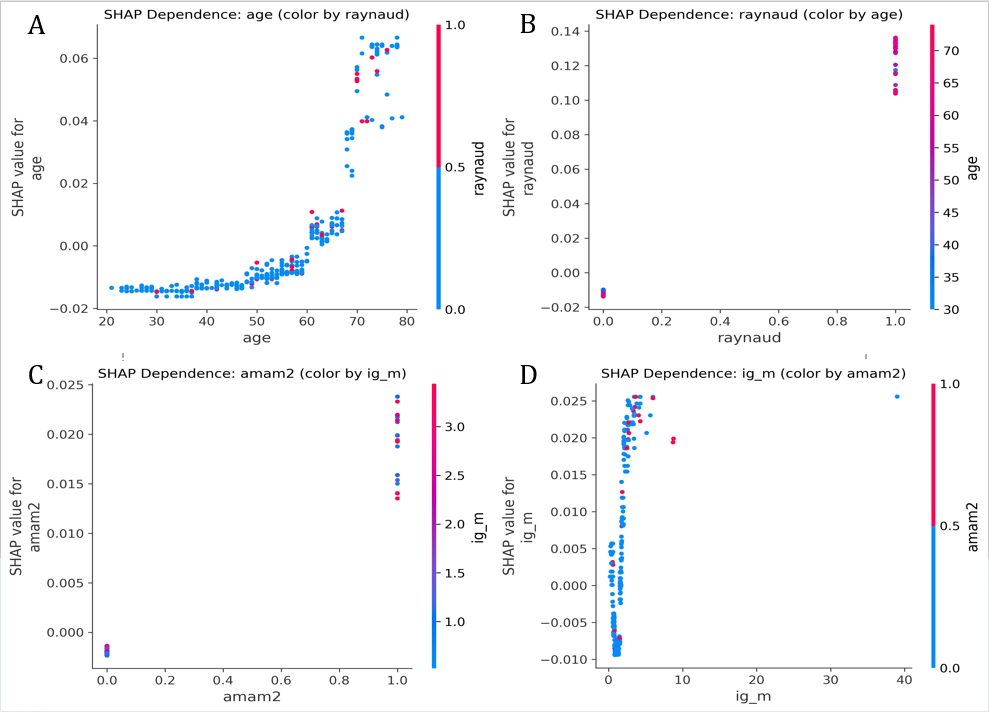

Supplement: Supplementary Figure 1 — Comparative calibration curves of the six evaluated machine learning algorithms in the validation cohort. [file Image1.tiff]

# Calibration Curve (Validation)

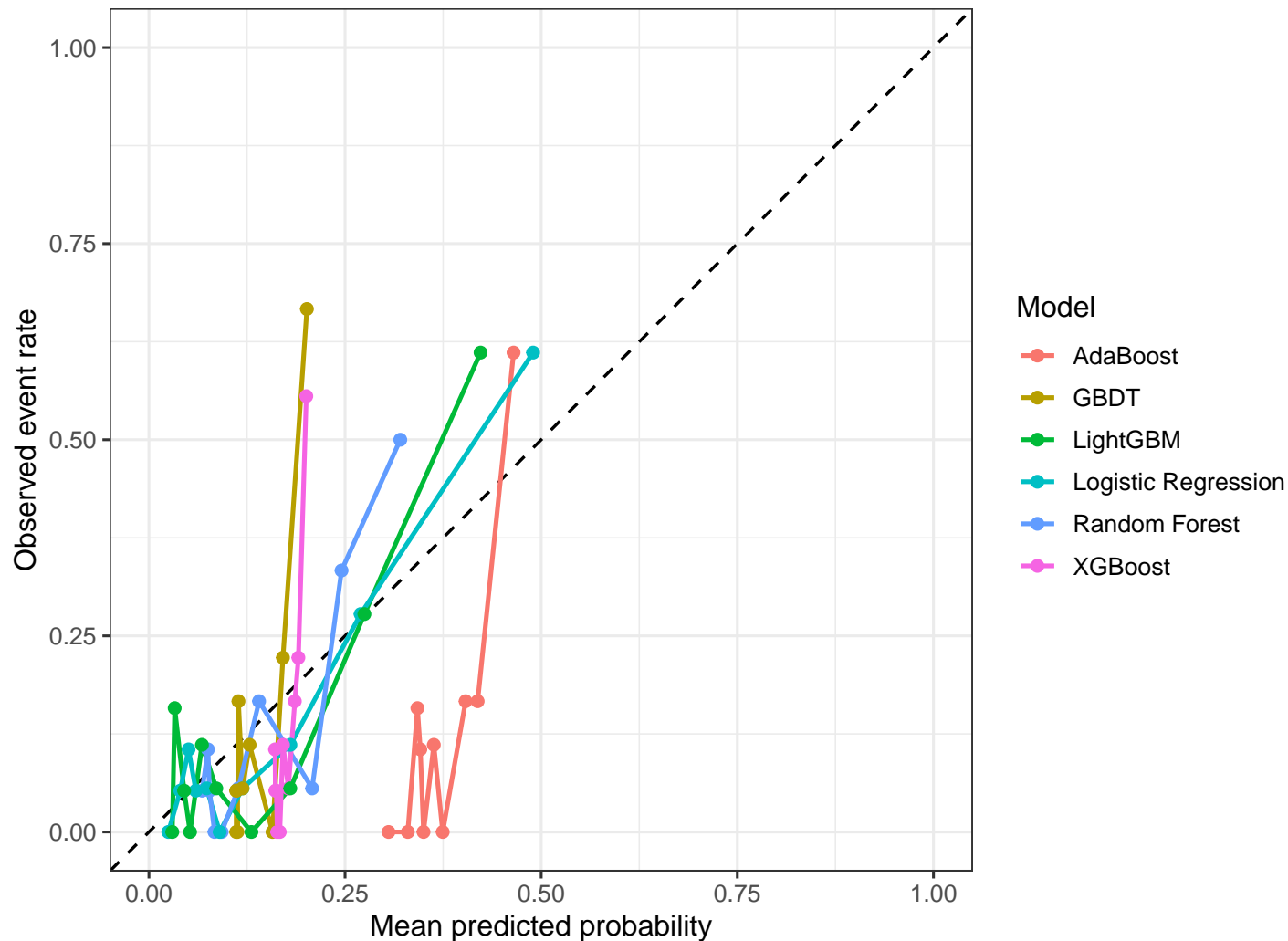

Supplement: Supplementary Figure 2 — Decision curve analysis (DCA) evaluating the clinical utility of the six machine learning models in the validation cohort. [file DataSheet1.pdf]

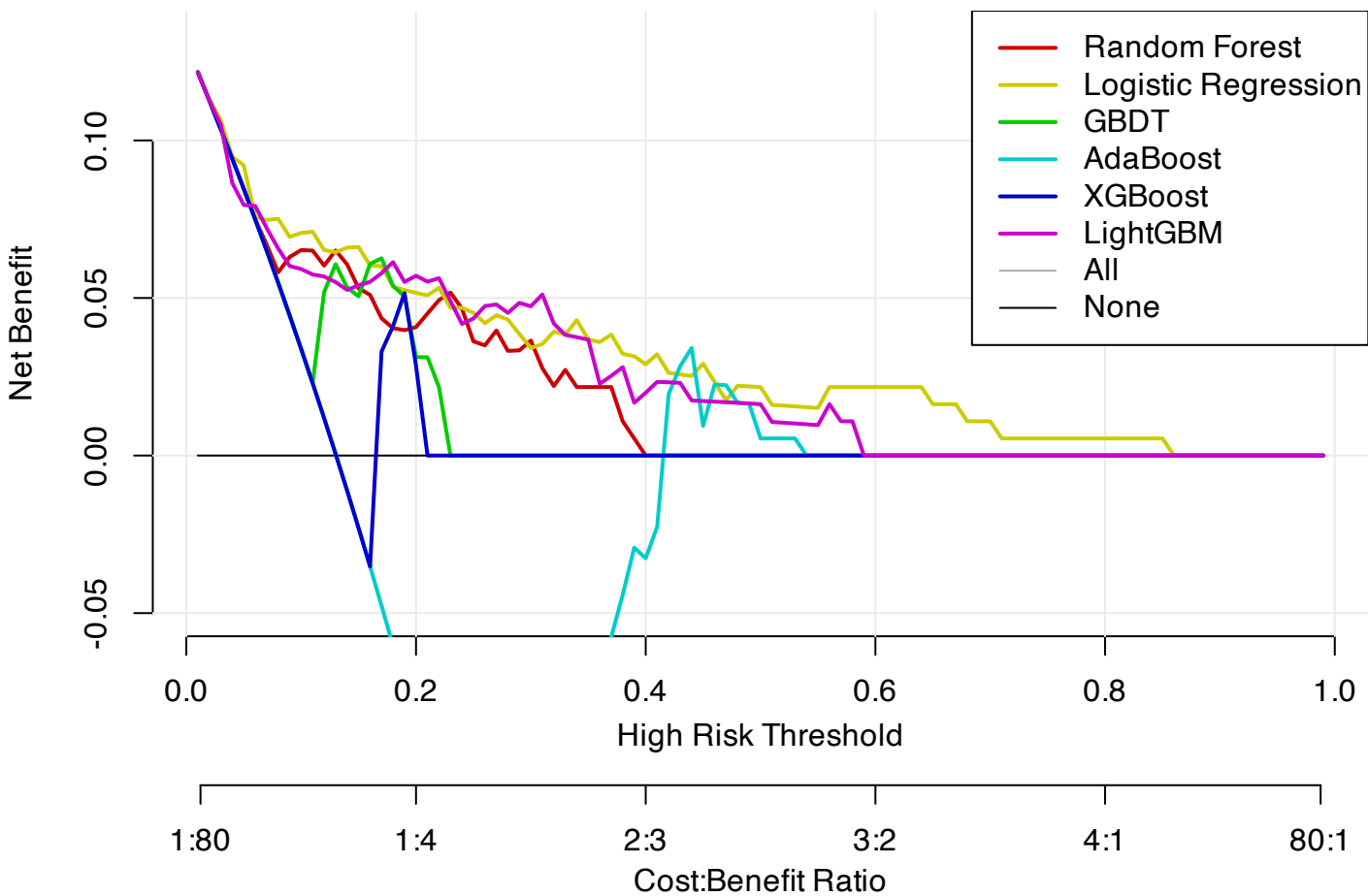

Supplement: Supplementary Figure 3 — SHAP dependence and interaction plots for key features. (A–B) SHAP interaction between RP and age. (C–D) SHAP interaction between anti-AMA-M2 positivity and serum IgM level. [file DataSheet2.pdf]
